# The main genetic locus associated with the evolution of gamecocks is centered on ISPD

**DOI:** 10.1093/g3journal/jkad267

**Published:** 2023-11-22

**Authors:** Andres Bendesky, Joseph Brew, Kerel X Francis, Enrique F Tello Corbetto, Antonio González Ariza, Sergio Nogales Baena, Tsuyoshi Shimmura

**Affiliations:** Department of Ecology, Evolution and Environmental Biology and Zuckerman Mind Brain Behavior Institute, Columbia University, New York, NY, 10027-2325, USA; Department of Ecology, Evolution and Environmental Biology and Zuckerman Mind Brain Behavior Institute, Columbia University, New York, NY, 10027-2325, USA; Department of Ecology, Evolution and Environmental Biology and Zuckerman Mind Brain Behavior Institute, Columbia University, New York, NY, 10027-2325, USA; Independent Researcher; PAIDI AGR-218 Research Group, Department of Genetics, Faculty of Veterinary Sciences, University of Córdoba, 14014 Córdoba, Spain; Diputación Provincial de Córdoba, Agropecuary Provincial Centre, 14014 Córdoba, Spain; PAIDI AGR-218 Research Group, Department of Genetics, Faculty of Veterinary Sciences, University of Córdoba, 14014 Córdoba, Spain; Department of Biological Production, Tokyo University of Agriculture and Technology, Fuchu, Tokyo 183-8509, Japan

**Keywords:** behavioral genetics, population genetics, domestication, evolution, animal behavior

## Abstract

Chickens were domesticated >4,000 years ago, probably first for fighting them and only later as a source of food. Fighting chickens, commonly known as gamecocks, continue to be bred throughout the world, but the genetic relationships among geographically diverse gamecocks and with nongame chickens are not known. Here, we sequenced the genomes of 44 geographically diverse gamecocks and 62 nongame chickens representing a variety of breeds. We combined these sequences with published genomes to generate the most diverse chicken genomes dataset yet assembled, with 307 samples. We found that gamecocks do not form a homogeneous group, yet they share genetic similarities that distinguish them from nongame chickens. Such similarities are likely the result of a common origin before their local diversification into, or mixing with nongame chickens. Particularly noteworthy is a variant in an intron of the *isoprenoid synthase domain containing* gene (ISPD), an extreme outlier present at a frequency of 89% in gamecocks but only 4% in nongame chickens. The ISPD locus has the strongest signal of selection in gamecocks, suggesting it is important for fighting performance. Because ISPD variants that are highly prevalent in gamecocks are still segregating in nongame chickens, selective breeding may help reduce its frequency in farm conditions in which aggression is not a desired trait. Altogether, our work provides genomic resources for agricultural genetics, uncovers a common origin for gamecocks from around the world and what distinguishes them genetically from chickens bred for purposes other than fighting, and points to ISPD as the most important locus related to fighting performance.

## Introduction

Chickens were one of the first animals to be domesticated, ∼4,000–10,000 years ago in South Asia, from the Red junglefowl (*Gallus gallus*) (Tixier-Boichard et al. 2011; Lawler 2012; Peters et al. 2016; Lawal et al. 2020; Wang et al. 2020b; Lawal and Hanotte 2021). They are now the most abundant land vertebrate, with a population size of over 33 billion and more than 25 billion chickens produced every year (FAO 2023). Today, chickens are by and large used for egg and meat consumption. Archaeologists propose, however, that chickens may have been first domesticated as gamecocks for fighting purposes and that this use drove their spread throughout the world (Tixier-Boichard et al. 2011; Perry-Gal et al. 2015; Lawler 2016; Luo et al. 2020). Evidence of their use for fights dates to at least 2,500–2,700 Bc in the Indus Valley (Tixier-Boichard et al. 2011) and China (Luo et al. 2020).

Organized chicken fights are still common and ingrained in cultures throughout the world (Geertz 1972; Dundes 1994). The chickens used in fights are distinct from those bred as a source of food; instead, they are selectively bred for fighting. Some gamecocks are bred as defined breeds, such as the Gallo Combatiente Español, the Gallo Navajero Peruano, the French Combattant du Nord, and varieties of Japanese Shamo, whereas others are more mixed. In some places, gamecocks are no longer used in fights but are still actively produced to maintain historical breeds, which can trace their origins to hundreds of years (Bixler 2000; Various authors 2005; Denegri 2015).

Gamecock breeds vary in many traits but share a key behavioral feature (Various authors 2005). Some breeds are specialized for endurance in lengthy, hours-long fights, whereas others fight much shorter fights, aided by different kinds of sharp instruments attached to their legs by people (Cockfight 2023). Gamecocks also vary drastically in their anatomy: there is a 350% range in their body weight, from the compact 2-kg Red Cubans (Various authors 2005) to the tall 7-kg Shamo (Tsudzuki et al. 2007). Thus, gamecocks vary in endurance, fighting style, body shape, and mass. By contrast, a behavioral feature shared by gamecocks, which sets them apart from nongame chickens, is their so-called “gameness”—a strong tendency to engage in a fight with another cock, rather than to flee, even after injury (Herzog 1979). Breeders directly select for gameness in behavioral tests before sanctioned fights, and indirectly by selectively breeding cocks (and their first-degree relatives) that have won fights (Cooper 1869; McIntyre 1906; Herzog 1979; Various authors 2005; Denegri 2015). The ultimate purpose of this strong selection for gameness and fight performance is to produce game chickens that will win fights.

There is consensus among gamecock breeders that nongame chickens are much less willing to engage in and persist in a fight than gamecocks (Denegri 2015 and personal communications from multiple breeders in Mexico, Peru, and Puerto Rico). Nevertheless, there are no scientific reports of the heritability or genetic bases of gameness. A recent genome-wide scan of selection in Chinese gamecocks identified multiple loci with low heterozygosity and that these loci genetically distinguish them from Chinese nongame chickens (Luo et al. 2020). However, genetic scans of selection in gamecocks outside China have not been performed, so it is unknown if the same or different loci are involved in gamecocks globally. Furthermore, how gamecocks from around the world are related to each other and to local and global nongame chickens is not known.

Although the history of chicken domestication suggests an ancient and common origin of gamecocks, it is possible that different gamecock breeds arose independently, from local chicken populations. Here, we set out to discover the genetic relationships among gamecocks from around the world, and to that end also assembled a phenotypically and geographically diverse set of samples from chickens bred for purposes other than fighting. We then searched for genetic similarities among gamecocks of different breeds and for genetic signatures of selection that would point to possible causes of their fighting performance.

## Methods

### Sample acquisition and whole genome sequencing

Animal protocols were approved by the Institutional Animal Care and Use Committee of Columbia University. We obtained blood samples after informed consent from breeders. We drew blood from the wing vein, deposited it in Whatman 903 cards, let dry completely, and then stored it in a ziploc bag at −20°C until DNA extraction. We extracted DNA from a 3.2-mm circular punch of the blood card by digesting in 5 µL of proteinase K with 195 µL of a detergent solution composed of 1% sodium dodecyl sulfate, 0.5% Tween 20, 1 mM ethylenediaminetetraacetic acid, 150 mM NaCl, and 50 mM Tris-Cl pH 7.5, followed by Omega Magbind Blood and Tissue kit. We then created Tn5-tagmented, 9-PCR cycles amplified, whole-genome sequencing libraries (Picelli et al. 2014) of an average insert size of 300–400 bp. We sequenced libraries with 2 × 150 bp reads in an Illumina Novaseq to a median depth (DP) of 12.2× (minimum 8×) after removing duplicate reads (see below).

Additionally, we downloaded sequencing data of 32 individuals with >9× coverage from the Chicken SD database, http://bigd.big.ac.cn/chickensd/. We also downloaded resequencing data of 300 samples with >8× coverage across all 4 *Gallus* species and 59 named chicken breeds from 12 NCBI BioProjects: PRJDB4092 (Ulfah et al. 2016), PRJEB39275 (Gheyas et al. 2021), PRJNA202483 (Fan et al. 2013), PRJNA232548 (Ulfah et al. 2016), PRJNA241474 (Wang et al. 2015), PRJNA306389 (Li et al. 2017), PRJNA432200 (Lawal et al. 2020), PRJNA482210 (Huang et al. 2020), PRJNA524911 (Noorai et al. 2019), PRJNA547951 (Wang et al. 2020a), PRJNA552722 (Guo et al. 2019), PRJNA597842 (Guo et al. 2022a), and PRJNA659069 (Sun et al. 2022).

### Variant calling and filtering

We followed genome analysis toolkit (GATK) best practices for mapping and cleaning up short read sequence data efficiently (Broad Institute 2023a): (1) we converted fastq files to uBAMs using picard FastqToSam (Broad Institute 2023b); (2) we marked adapters (NEXTERA_V2) using picard MarkIlluminaAdapters; (3) we aligned reads to the bGalGal1.mat.broiler.GRCg7b (GCF_016699485.2, part of the Vertebrate Genomes Project (Rhie et al. 2021)) *Gallus gallus* reference genome by piping picard SamToFastq output to bwa mem2 (Vasimuddin et al. 2019) and piped the output to picard MergeBamAlignment; and (4) we merged alignments of the same individual coming from multiple sequencing runs and marked duplicate reads using picard MarkDuplicates.

We called variants using bcftools mpileup (Danecek et al. 2021) and then filtered individual calls and then sites from all individuals using bcftools according to the following criteria. For individual calls, we included only variants >3 bp from an indel, that had an MQ≥50.0 and MQ0F≤0.1, excluded indels and variants with more than 2 alleles, included only autosomal variants whose DP was <1.65× the average autosomal DP for that sample (Bergström et al. 2020), excluded variants with DP < 4 GQ < 30, that were heterozygous yet AD < 2 for either allele, that were heterozygous yet the AD ratio of one allele to the other was <0.25 or >0.75. For sites, we excluded those that had missing genotypes (according to the filtering criteria above) in more than 20% of samples. We also excluded sites that fell in poor mapability regions according to a snpable (Li 2009) mask of the bGalGal1 genome ran with the following parameters: kmer length k = 100, map with bwa aln -t 16 -R 1,000,000 -O 3 -E 3, stringency r = 0.5.

### Sample filtering and quality control

We checked the sequencing quality of each sample using fastp (Chen et al. 2018). We excluded downloaded sample SRR1217514 because over 99% of the reads had low quality (average read quality score < Q15). We further removed samples from the downloaded dataset that did not have >7.5× coverage after duplicates had been removed. The median depth of the remaining downloaded samples was 12.6×. We also ensured that no 2 samples were identical using picard crosscheck fingerprint, nor were more closely related than first cousins using ngsRelateV2 (Hanghøj et al. 2019), nor showed signs of cross-contamination based on variant allele frequencies >20% across the mitochondrial genome. Finally, we removed samples that had more than 30% genotypes missing after all the site and genotype filters above had been applied. An additional published Red junglefowl sample (SRR1217528) was removed because it clustered with domesticated chickens rather than with all other Red junglefowl in both the genome-wide phylogenetic tree and in principal component analysis (PCA), suggesting this was a Red junglefowl–chicken hybrid or the result of sample contamination.

### Phylogenetic analyses

For the genome-wide phylogenetic tree, we sampled ∼0.1% of the genome by selecting 1,000 nonoverlapping 10-kb regions randomly distributed throughout the genome except the Z and W chromosomes, and generated multifasta files from a bcf file containing invariant sites. Using this multifasta file, we generated a genome-wide maximum likelihood tree using IQTree v2.0.3 (Kalyaanamoorthy et al. 2017; Hoang et al. 2018; Minh et al. 2020) with the following parameters: -t PARS -ninit 2 -bb 1000 -nm 1000 -m TEST (to find the best-fit substitution model for the data, which was HKY + F + I). For the phylogenetic tree of the Chromosome 2 population branch statistic (PBS) (26.6–28.2 Mb) and GWAS (28,061,838–28,260,724 bp) peaks, we ran IQTree on fasta files including invariant sites of the regions using the following parameters: -t PARS -ninit 2 -bb 1000 -nm 5000 -m HKY + F + I. We plotted the trees using toytree 2.0.5 (Eaton 2020).

### Principal component analysis and ADMIXTURE

For genetic PCA, we used PLINK v1.90b6.21 (Chang et al. 2015; Purcell and Chang 2022) on autosomal variants with a minimum allele count of 1 with the following parameters for linkage disequilibrium pruning of variants –indep-pairwise 50 kb window size 10 kb step size 0.2 r^2^ threshold. We plotted the results using seaborn (Waskom 2021). We ran ADMIXTURE v1.3 (Alexander et al. 2009) at K = 1–8 using the plink linkage-disequilibrium pruned variants at 0.1 r^2^ threshold and present the results for K = 8 because it had the lowest cross-validation error.

### Genome-wide association analysis

We performed genome-wide association analysis (GWAS) comparing gamecocks (n = 48) to nongame chickens (n = 62). We restricted the analysis to samples we were confident in their breeding for fighting or other purposes: those we collected specifically for this study plus the 4 published Chinese gamecock samples that passed our filtering criteria and quality control. We used the 9,023,221 autosomal variants at a minimum allele frequency of 5%. To control for population stratification, we first clustered the samples using complete linkage agglomerative clustering based on pairwise identity-by-state distance, using PLINK v1.90b6.21. Both K = 4 (ƛ = 2.61) and K = 5 (ƛ = 2.61) led to the lowest genomic inflation compared to K = 2 (ƛ = 5.24), 3 (ƛ = 4.60), 6 (ƛ = 4.51), 7 (ƛ = 4.53), 8 (ƛ = 4.42), 9 (ƛ = 4.32), 10(ƛ = 4.23). However, K = 4 included more samples in clusters than K = 5 so we performed association testing using K = 4, accounting for clusters, and calculated statistical significance using genomic control.

--cluster --K 4 --allow-extra-chr --allow-no-sex --out out

--mh --within out.cluster2 --adjust --allow-extra-chr --allow-no-sex --out aac

We then performed 10,000 permutations, also accounting for clusters with K = 4, and used a permutation-based significance threshold of *P* < 10^−4^.

### Population branch statistic, heterozygosity, and nucleotide diversity

We calculated PBS (Yi et al. 2010), with the focal population being Japanese gamecocks, and the Japanese nongame chickens and Ethiopian nongame chickens as the contrasts. We used variants with a minimum allele count of 1. PBS was calculated in windows of 1,000 variants with 200 step size, using scikit-allel v.1.3.5 (Miles 2021).

Heterozygosity was calculated separately in gamecocks and nongame chickens (as described in GWAS section), in windows of 10,000 variants and 1,000 step size, using scikit-allel v.1.3.5.

Nucleotide diversity within the PBS peak region (Chromosome 2: 26.6–28.2 Mb) was calculated using pixy v1.2.7beta1 (Korunes and Samuk 2021; Samuk et al. 2022) on a vcf file that included invariant sites, separately in gamecocks and nongame chickens (as described in GWAS section).

### Phasing

To plot haplotypes, we first phased genotypes using SHAPEIT5 v5.1.0 (Hofmeister et al. 2023) phase_common using default parameters.

## Results

To study the genetics of gamecocks, we began by collecting DNA samples of 44 fighting chickens from around the world and sequencing their genome to ∼12× coverage. After combining them with 4 other published gamecock genomes, this dataset includes chickens representing 15 countries and 29 breeds (Table 1). As a comparison group of chickens bred for purposes other than fighting, we further sequenced 62 nongame chickens and used 181 additional published nongame chicken samples (Supplementary Table 1). Together, the set includes samples from 12 countries and 108 recognized chicken breeds, or chickens bred in a particular location but without a particular breed name. Many of these breeds had never been sequenced. This set of samples thus constitutes the most diverse collection of chicken genomes sequenced at relatively high coverage.

**Table 1.** Gamecock genomes sequenced and analyzed in this study.

Sample origin	Breed	Number of samples	Source
Bali	Balinese	1	This study
Brazil	Shamo	1	This study
China	Luxi game	1	Ulfah et al. (2016)
China	Xishuangbanna game fowl	3	Wang et al. (2015)
Colombia	Pico	1	This study
France	Combattant du Nord	1	This study
Japan	Koshamo-Fukushima	1	This study
Japan	Nankinshamo-Hiroshima	1	This study
Japan	Oshamo	14	This study
Japan	Tauchi	1	This study
Japan	Yakido-Fukushima	1	This study
Japan	Yakido-Hyogo	1	This study
Japan	Yakido-Mie	1	This study
Malaysia	Omkoi	1	This study
Mexico	Hatch	1	This study
Mexico	Mixed	1	This study
Mexico	Navajero	1	This study
Mexico	Sweater	1	This study
Pakistan	Asil	1	This study
Peru	Navajero Peruano	1	This study
Peru	Pico	2	This study
Puerto Rico	Pico	1	This study
Spain	Combatiente Español	1	This study
Spain	Combatiente Canario	1	This study
Thailand	Nakhon Sawan	1	This study
USA	Black Breasted Red Aseels	1	This study
USA	Blue Sumatra	1	This study
USA	Dark Aseel	1	This study
USA	Sumatra	1	This study
USA	Wheaton Aseel	1	This study
Vietnam	Saigon	1	This study

### Gamecocks do not constitute a homogeneous group

**Fig. 1.**
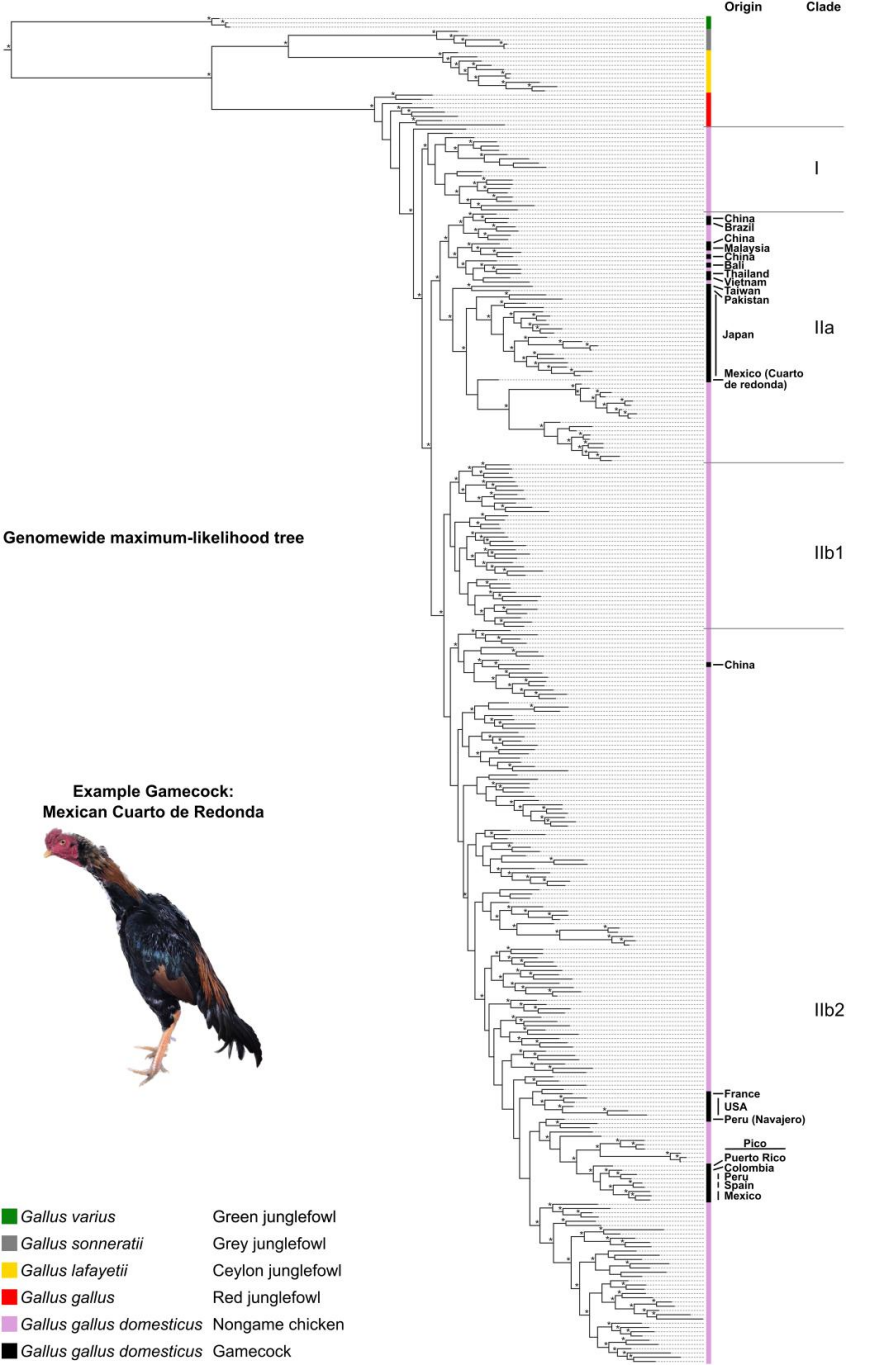
Phylogenetic tree of *Gallus* including chickens. Maximum-likelihood phylogenetic tree based on whole-genome data, including all species in the junglefowl (*Gallus*) genus, as well as gamecocks and nongame chickens from around the world. The geographic origin of gamecock breeds is highlighted on the right. *, bootstrap support values ≥80. Supplementary Fig. 1 is a zoomable and text-search friendly version of the figure showing the location and name of all samples as well as the bootstrap support values.

To determine whether gamecocks from around the world are more closely related to each other than to chickens not bred for fighting, we built a phylogenetic tree based on whole genome data of the 48 gamecock samples and the 243 nongame domesticated chickens (Fig. 1 and Supplementary Fig. 1). To build this tree, we also included published genomes of Red junglefowl (*Gallus gallus*), from which the chicken was domesticated (Fumihito et al. 1996; Lawal et al. 2020; Wang et al. 2020b; Lawal and Hanotte 2021), as well as from the closely related Ceylon junglefowl (*Gallus lafayetii*), the Gray junglefowl (*Gallus sonneratii*), and, as an outgroup, the more distantly related Green junglefowl (*Gallus varius*). As expected, the Green junglefowl formed an outgroup to all other samples. The Ceylon and Gray junglefowls appear as sister species to each other and are more closely related to Red junglefowls than to Green junglefowls, in agreement with previous observations (Fumihito et al. 1996; Wang et al. 2020b). All chickens, including gamecocks, are included in a monophyletic branch that is distinct but closest to the Red junglefowls, consistent with chickens being domesticated from the Red junglefowl.

Chickens separate into 2 major clades: I and II. Clade I contains exclusively nongame chickens from China. Clade II is further divided into IIa and IIb. IIa contains almost exclusively Asian nongame and game chickens as well as a gamecock sample from Mexico and one from Brazil; the breeders of these Latin American gamecocks acknowledged using Asian gamecocks to produce these individuals, likely explaining their phylogenetic placement. Clade IIb is subdivided into 2 further clades. Clade IIb1 is composed exclusively of nongame chickens from China. By contrast, Clade IIb2 is the most diverse group, including samples from most locations. All the European, African, and New World game and nongame chickens (except the Mexican and Brazilian ones noted above) are contained in this clade. This clade also contains chickens from East Asia and Iran. Because mixed background might differ among chickens of a given type or location, and because many of the chicken varieties we sampled are represented by a single individual, the phylogenetic tree should not be taken as a definitive relationship between chicken varieties. Still, the results show that chicken genetic relatedness is broadly geographically structured, but with some exceptions likely originating from recent mixing with animals imported from other locations.

Notably, gamecock samples do not all cluster together but rather are interspersed with nongame chickens, often with individuals from the same geographic region (Fig. 1 and Supplementary Fig. 1). The lack of a single gamecock cluster suggests that gamecocks have independent local origins or a common origin followed by mixing with local populations.

### Phylogenetic patterns reflect historical events

The patterns of relationships among gamecocks appear to parallel their history. Fighting chickens from North America, South America, and the Caribbean are most closely related to the Combatiente Español and Combatiente Canario gamecock breeds from Spain, consistent with the introduction of gamecocks into the New World by the Spanish (Bixler 2000; Lawler 2016) (Supplementary Fig. 1). These Hispanic and New World gamecocks are most closely related to the Leghorn (Livornese) chickens that originated in Tuscany, possibly related to an introduction of gamecocks into Spain by the Romans (Various authors 2005; Lawler 2016). The close relationship between Hispanic gamecocks and Italian nongame chickens indicates these breeds share more ancestry than other breeds.

### Gamecocks share genetic similarities

**Fig. 2.**
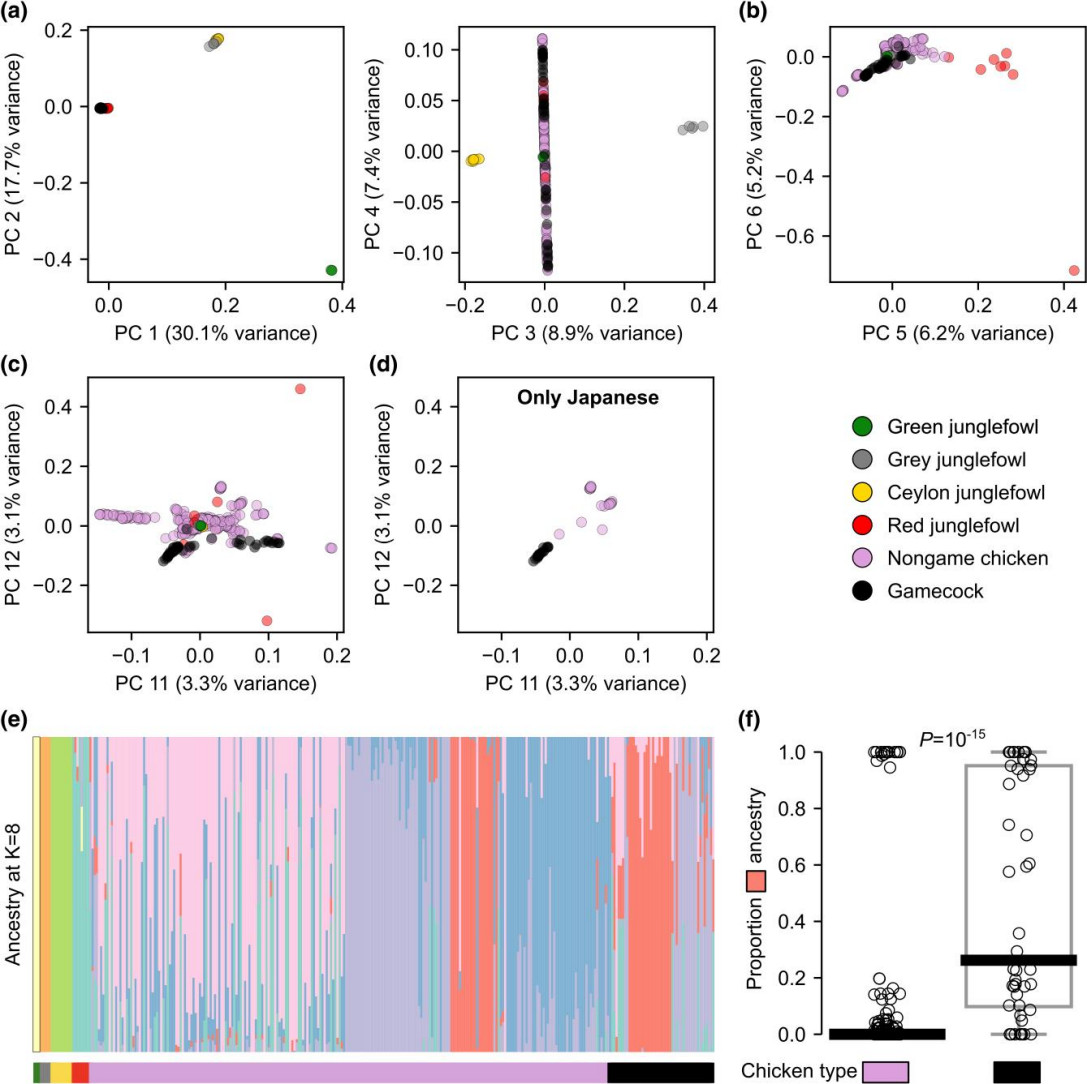
*Gallus* genetic structure. a-d) PCA of genetic variation in *Gallus* samples. a) PC 1–3 separate samples by species. b) PC 5 separates *Gallus gallus* into Red junglefowl and domesticated chickens. c) PC 12 mostly separates chickens into gamecocks and nongame chickens. d) Same as c but showing Japanese samples exclusively. e) ADMIXTURE analysis at K = 8 (which had the lowest cross-validation error, see Supplementary Fig. 3a). f) Proportion of “salmon-colored” ancestry from e in nongame chickens (n = 243) and gamecocks (n = 48). *P*-value by Mann-Whitney *U* test.

The genome-wide phylogeny indicated that gamecocks do not form a homogeneous group. Nonetheless, gamecocks may still share genetic ancestry distinct from nongame chickens in a fraction of their genome, indicative of a common origin. To test this possibility, we began by performing PCA of genome-wide genetic variation. The first 3 principal components (PCs) cleanly separated samples by species (Fig. 2a). Gamecocks clustered closely with nongame chickens and with Red junglefowl in the first 2 PCs (Fig. 2a), suggesting no large-scale hybridization of any of these *Gallus gallus* samples with other *Gallus* species, in agreement with the genome-wide phylogeny. PC 5 largely separated *Gallus gallus* into domesticated and wild individuals (Fig. 2b and Supplementary Fig. 2a). In turn, PC 12 largely separated domesticated chickens into game and nongame (Fig. 2c and Supplementary Fig. 2b). Remarkably, this PC also separated Japanese chickens into game and nongame (Fig. 2d and Supplementary Fig. 2c), even though these 2 groups are otherwise closely related and form sister branches in the genome-wide phylogeny (Supplementary Fig. 1). These results indicate that gamecocks from around the world share genetic similarities.

Consistent with these findings, an ADMIXTURE analysis to characterize genetic ancestry components revealed that gamecocks have significantly (*P* = 10^−15^ by a Mann-Whitney U test) more of a particular ancestry (median of 26%) than do nongame domesticated chickens (median of 0%) (Fig. 2, e and f and Supplementary Fig. 3a). This “game ancestry” was not driven by the Japanese samples, which constitute 15% of the dataset, as the game ancestry remains significantly (*P* = 10^−10^) more common in non-Japanese gamecocks than in non-Japanese nongame chickens (Supplementary Fig. 3b). The game ancestry does not appear to correspond to that of closely related *Gallus* species, suggesting that it is unlikely the product of incomplete lineage sorting or gene flow from those species (Fig. 2e). Thus, although gamecocks do not form a homogeneous phylogenetic group, they share a significant component of their ancestry. Even though gamecocks had significantly more of this game ancestry, some gamecocks lacked it and some nongame chickens had substantial game ancestry, suggesting that only a fraction of this ancestry might be relevant for fighting-related traits. A parsimonious explanation for these observations is that gamecocks from around the world had a common origin, which was followed by local diversification into or local mixing with nongame chickens, but that some of their original game ancestry—perhaps that which is most relevant for fighting performance—was maintained through selective breeding, despite local mixing.

### Gamecocks share genetic variants under selection

**Fig. 3.**
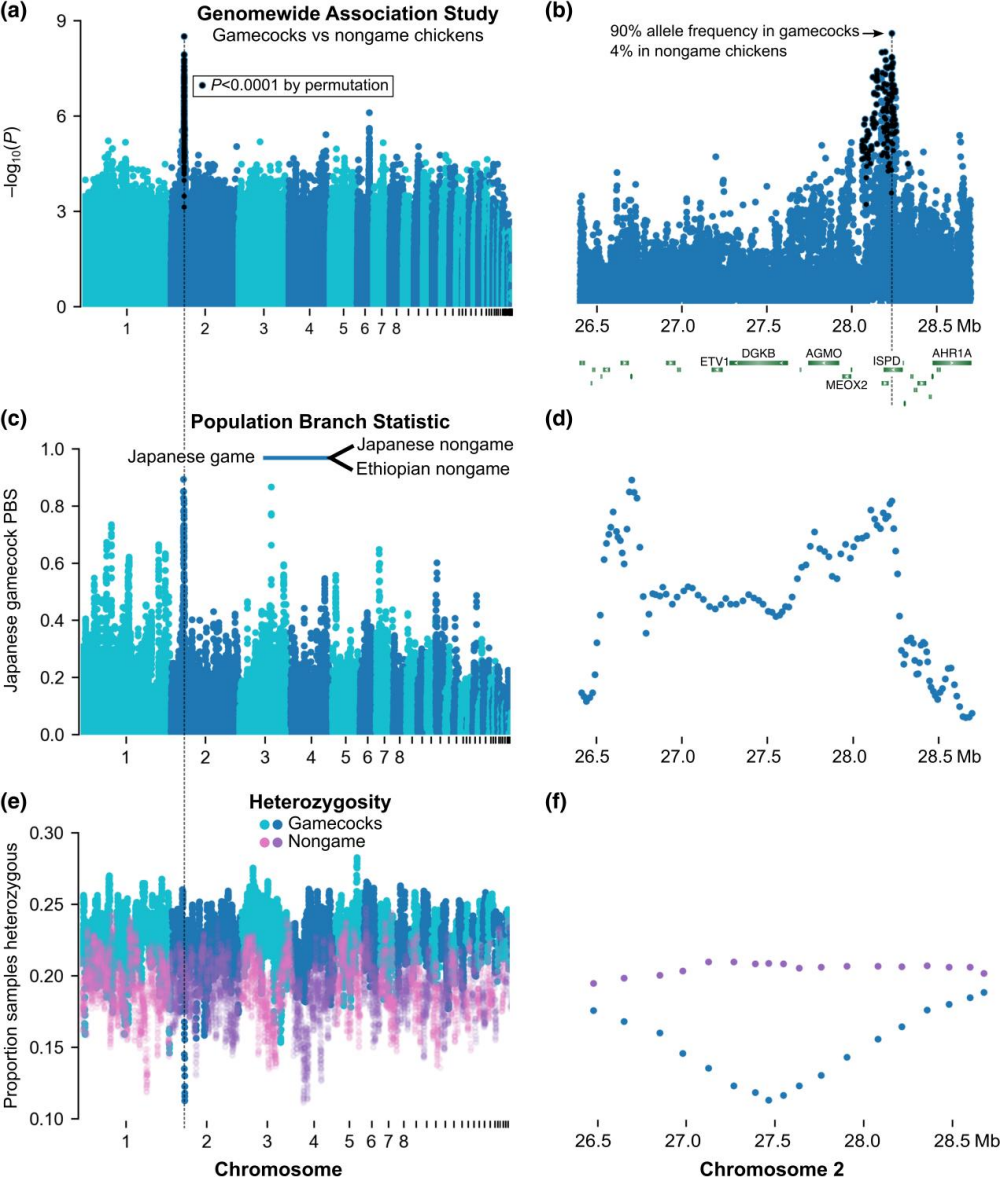
Genetic loci that distinguish gamecocks from nongame chickens are under selection. a) GWAS comparing gamecocks (n = 48) to nongame chickens (n = 62) and b) zoom of top hit. *P*-values on the y-axis using genomic control. Black denotes variants with *P* < 10^–4^ by permutation; no variants outside chromosome 2 surpassed that permutation threshold. c and d) Windowed PBS comparing Japanese gamecocks (n = 14) to Japanese nongame chickens (n = 19) and Ethiopian nongame chickens (n = 34). The Japanese gamecock branch statistic is shown on the y-axis. e and f) Windowed heterozygosity in gamecocks (n = 48) and nongame chickens (n = 62).

To identify genetic loci shared by gamecocks, we performed a genome-wide association study (GWAS) comparing gamecocks to nongame chickens, using hierarchical clustering to control for population structure (Purcell et al. 2007) (Fig. 3a and Supplementary Fig. 4b). We found a particularly prominent peak in Chromosome 2, near the genes *alkylglycerol monooxygenase* (AGMO), *mesenchyme homeobox 2* (MEOX2), an uncharacterized RNA with an open reading frame, and reaching the zenith in an intron of *isoprenoid synthase domain containing* (ISPD) (Fig. 3b). Intriguingly, this locus was recently identified in an independent study as the most differentiated (highest *F*_ST_) genomic region between Chinese gamecocks and Chinese nongame chickens (Luo et al. 2020). The GWAS peak we identified is not driven by Chinese chicken samples, since they only constitute 8% of our game samples and none of our nongame samples, and the peak remains the top hit when excluding Chinese chickens (Supplementary Fig. 4, a and c). Thus, this Chromosome 2 locus is the genetic region that most distinguishes gamecocks from nongame chickens worldwide.

The most significantly (Genomic Control *P* = 3 × 10^−9^; permutation *P* < 10^−4^) associated variant (rs735074467), at position 28,236,213 in Chromosome 2 within an intron of the ISPD gene, is present at a frequency of 3.7% in nongame chickens but 89.5% in gamecocks (Fig. 3b). By contrast, the ISPD missense variant Arg84Lys that was previously identified as a likely causal variant in Chinese gamecocks (Luo et al. 2020) is present at a frequency of 3.3% in nongame chickens but only 37.0% in gamecocks and it does not reach genome-wide significance (Genomic Control *P* = 1.7 × 10^−3^; permutation *P* = 1) in our GWAS. The intronic and missense variants are in low linkage disequilibrium (*r^2^* = 0.22), allowing for a larger and more diverse gamecock sample, such as ours, to determine which variant has a stronger association with gamecocks. Thus, noncoding variation within ISPD is a more likely genetic cause of differences between gamecocks and nongame chickens worldwide.

If gamecocks had a common origin and later mixed with nongame chickens, gamecock ancestry could be diluted randomly, in which case the Chromosome 2 gamecock-associated variants remain in most gamecocks by chance. Alternatively, the Chromosome 2 locus variants might persist in gamecocks through the continued effects of selective breeding. In support of the selective breeding hypothesis, a population-branch statistic (PBS) analysis indicates that the Chromosome 2 locus is the part of the genome most differentiated in Japanese gamecocks compared to closely related Japanese nongame chickens and Ethiopian nongame chickens as a more distant control population, consistent with selection at the locus (Fig. 3, c and d). In line with the PBS results, the Chromosome 2 locus (the 1.6 Mb plateau with high PBS) has 38% lower nucleotide diversity in gamecocks (0.1% per site) than in nongame chickens (0.16%); and, notably, the locus has the lowest heterozygosity of the whole genome in gamecocks from throughout the world, but has unremarkable heterozygosity in nongame chickens, consistent with selection in gamecocks (Fig. 3, e and f). This region also has unusually low heterozygosity in Chinese gamecocks (Luo et al. 2020). Altogether, our results indicate that the Chromosome 2 locus is the region that most strongly distinguishes gamecocks from nongame chickens, likely because of the effects of selective breeding. Additional minor loci, with weaker signals of selection, could also contribute to gameness.

**Fig. 4.**
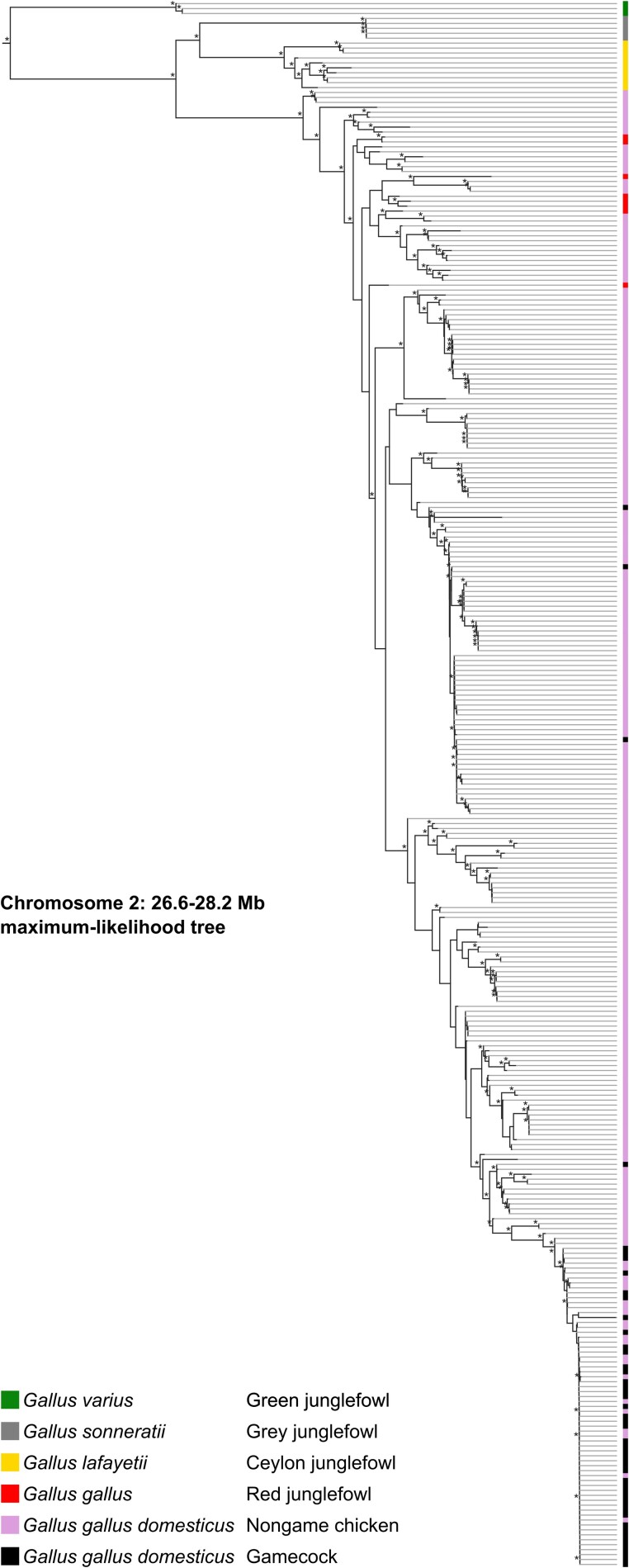
Phylogenetic tree of Chromosome 2 locus that distinguishes gamecocks from nongame chickens. Maximum-likelihood phylogenetic tree of Chromosome 2 locus (26.6–28.2 Mb), including all species in the junglefowl (*Gallus*) genus, as well as gamecocks and nongame chickens from around the world. *, bootstrap support values ≥90. Supplementary Fig. 5 is a zoomable and text-search friendly version of the figure showing the location and name of all samples as well as the bootstrap support values.

Because gamecocks share genetic ancestry, it is likely that the Chromosome 2 locus of gamecocks has a common origin, i.e. this region is identical by descent among them. Alternatively, the locus evolved independently and convergently in gamecocks. Consistent with a common origin, 92% (44/48) of gamecocks, including those from clades IIa and IIb1, cluster together in a branch of a phylogenetic tree of the 1.6-Mb Chromosome 2 PBS locus (Fig. 4 and Supplementary Fig. 5). By contrast, only 9% (21/243) of nongame chickens are in this grouping (*P* = 10^−31^ by Fisher's exact test). Very similar phylogenetic patterns are observed in a narrower 200-kb region encompassing the GWAS peak (Supplementary Fig. 6). In agreement with the phylogenetic clustering, gamecock and nongame haplotypes are very different (Supplementary Fig. 7). Thus, although gamecocks differ substantially from each other genome-wide (Fig. 1), they are very similar at the Chromosome 2 locus. All junglefowl samples follow the species tree at this locus, suggesting that this gamecock haplotype is not the result of introgression from another junglefowl species. Thus, the locus that is most associated with gamecocks has a common origin across gamecocks.

## Discussion

We assembled the most diverse panel of gamecocks and nongame chickens, of the Red junglefowl (the wild ancestor of the domesticated chicken), and of the other 3 species in the junglefowl (*Gallus*) genus. Through the analysis of whole genomes, we found that gamecocks from around the world (Bali, Brazil, China, Colombia, France, Japan, Malaysia, Mexico, Pakistan, Peru, Puerto Rico, Spain, Taiwan, Thailand, USA, and Vietnam) share common ancestry that is largely absent from nongame chickens. This pattern is likely the result of positive selection for high aggression in gamecocks and against aggression in nongame chickens, in which it is an undesirable trait (Nielsen et al. 2023).

Genome-wide, however, gamecocks do not form a homogeneous group. One possibility is that chickens were repeatedly and independently selectively bred for fighting around the world and parallel selection on standing variation increased the frequency of “gamecock ancestry” in geographically disperse chickens. Alternatively, gamecocks could have had a common origin and later mixed with local nongame populations as they dispersed through the world. The mixing with local populations could have diluted the “gamecock ancestry” through the random effects of recombination and the independent assortment of chromosomes at meiosis, such that specific loci would now be enriched in gamecocks only by chance. However, we find a locus on Chromosome 2, peaking at the gene ISPD, which strongly differs in variant frequencies between gamecocks and nongame chickens from around the world. In Japan, the “game” allele is present at 97% frequency in gamecocks and 10% in nongame chickens; outside Japan, it is present at 83% in gamecocks but not present in any of the 40 nongame chickens we sampled. This genetic region has been found to distinguish Chinese gamecocks from Chinese nongame chickens (Luo et al. 2020); our results now show that this locus is the most distinguishing genomic locus for gamecocks worldwide and provides a much finer resolution. Moreover, we find evidence for this locus being under selection in gamecocks, as it is particularly differentiated in gamecocks relative to the rest of the genome and shows the lowest levels of heterozygosity in the genomes of gamecocks but not in nongame chickens. Consistent with a common gamecock origin, this locus, in contrast to the rest of the genome, is very similar in most gamecocks.

Because gameness and fighting ability distinguish gamecocks from nongame chickens more than body size or other anatomical features, it is likely that the Chromosome 2 locus contributes to the behavior of gamecocks. It is possible, however, that it affects other traits that modulate fighting performance, such as athleticism. Previous reports focusing on Chinese gamecocks pointed to a missense (Arg84Lys) variant in ISPD within the Chromosome 2 locus as likely causal and that this variant might affect muscle traits (Luo et al. 2020; Guo et al. 2022b). However, our results, which encompass more geographically and genetically diverse gamecock and nongame chicken samples, as well as a larger sample size, indicate the ISPD missense variant is not significantly more frequent in gamecocks. Instead, our results point to an intronic variant in ISPD as the most pronounced genetic distinction between gamecocks and nongame chickens. Although genetic variation in and near ISPD may act by affecting other genes, ISPD itself is an appealing candidate not only because it's the gene closest to the peak of association, but also because ISPD is involved in axon guidance during the wiring of the central nervous system and in the development of the cerebral cortex (Wright et al. 2012). Regulatory variation at ISPD may therefore affect fighting performance through behavioral effects rather than, or in addition to, its effects on muscle.

Altogether, this work characterizes the commonalities and differences among the genomes of gamecocks and points to a locus in Chromosome 2 as a genetic factor under selection that strongly distinguishes gamecocks from other chickens. Because genetic variants associated with gamecocks are still segregating in nongame chickens, it might be possible to use these markers to select chickens with reduced aggression for breeding under conditions in which aggression is not a desirable trait.

## Supplementary Material

jkad267_Supplementary_Data

## Data Availability

Sequence data is available at NCBI Sequence Read Archive BioProject ID PRJNA973554. Code is provided in the supplement as Jupyter Notebooks (Supplementary File 1). Supplemental material available at G3 online.
