# Supplementary material for: The main genetic locus associated with the evolution of gamecocks is centered on ISPD
Source: G3 (Bethesda). 2023 Nov 22;14(2):jkad267. doi: 10.1093/g3journal/jkad267 (PMC10849328; doi:10.1093/g3journal/jkad267)
Supplement: jkad267_Supplementary_Data [file jkad267_supplementary_data.zip › Supplemental_Figures_G3-2023-404662.pdf]

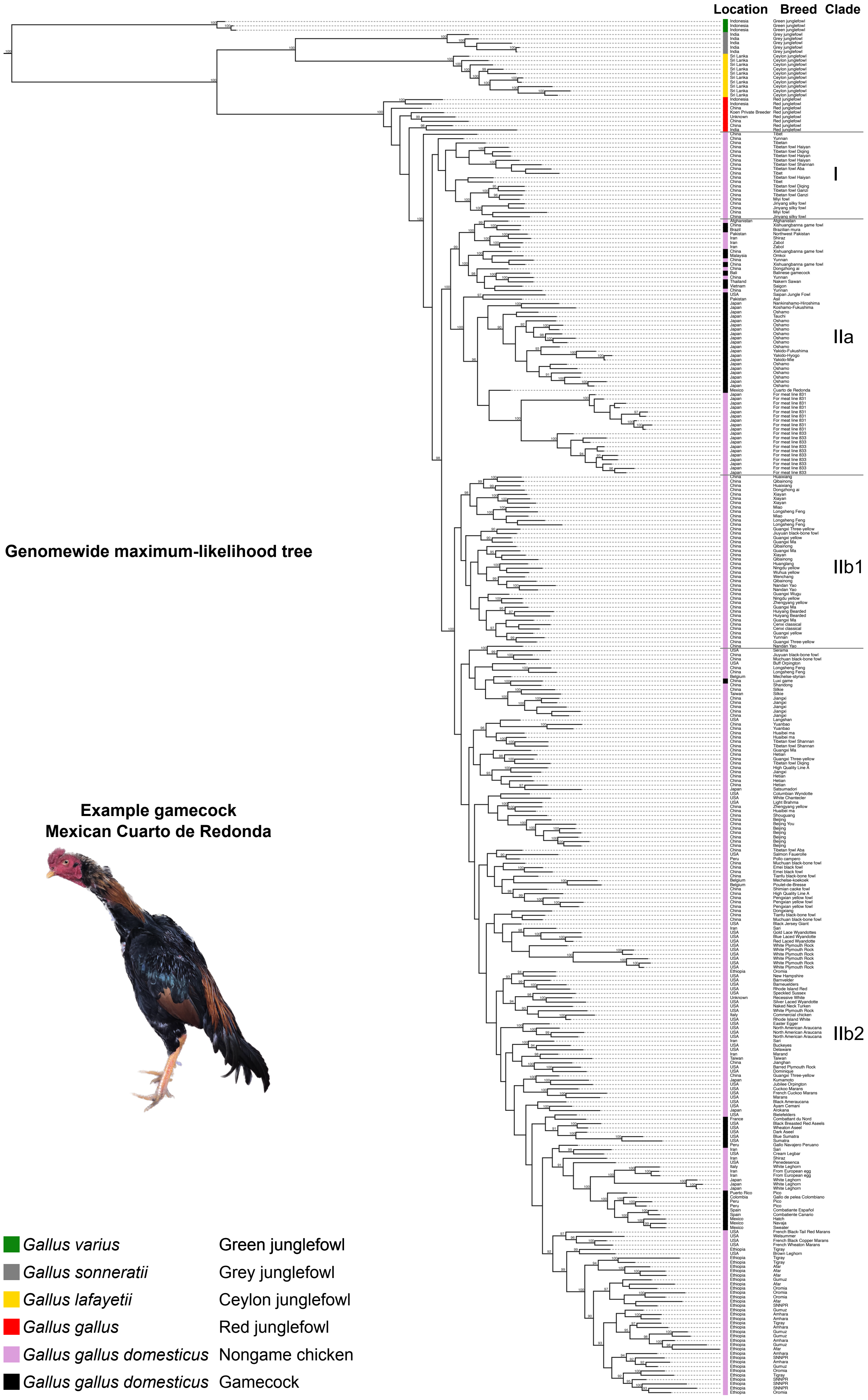
Location
Breed
Clade
I
IIa
IIb1
IIb2
Genomewide maximum-likelihood tree
Example gamecock
Mexican Cuarto de Redonda
Gallus varius
Green junglefowl
Gallus sonneratii
Grey junglefowl
Gallus lafayetii
Ceylon junglefowl
Gallus gallus
Red junglefowl
Gallus gallus domesticus
Nongame chicken
Gallus gallus domesticus
Gamecock

Figure S1. Genome-wide phylogenetic tree of *Gallus* including chickens

Maximum-likelihood phylogenetic tree based on whole-genome data, including all species in the junglefowl (*Gallus*) genus, as well as gamecocks and nongame chickens from around the world. The name and geographic origin of the samples is presented on the right. Bootstrap support values ≥90 are highlighted on the branches.

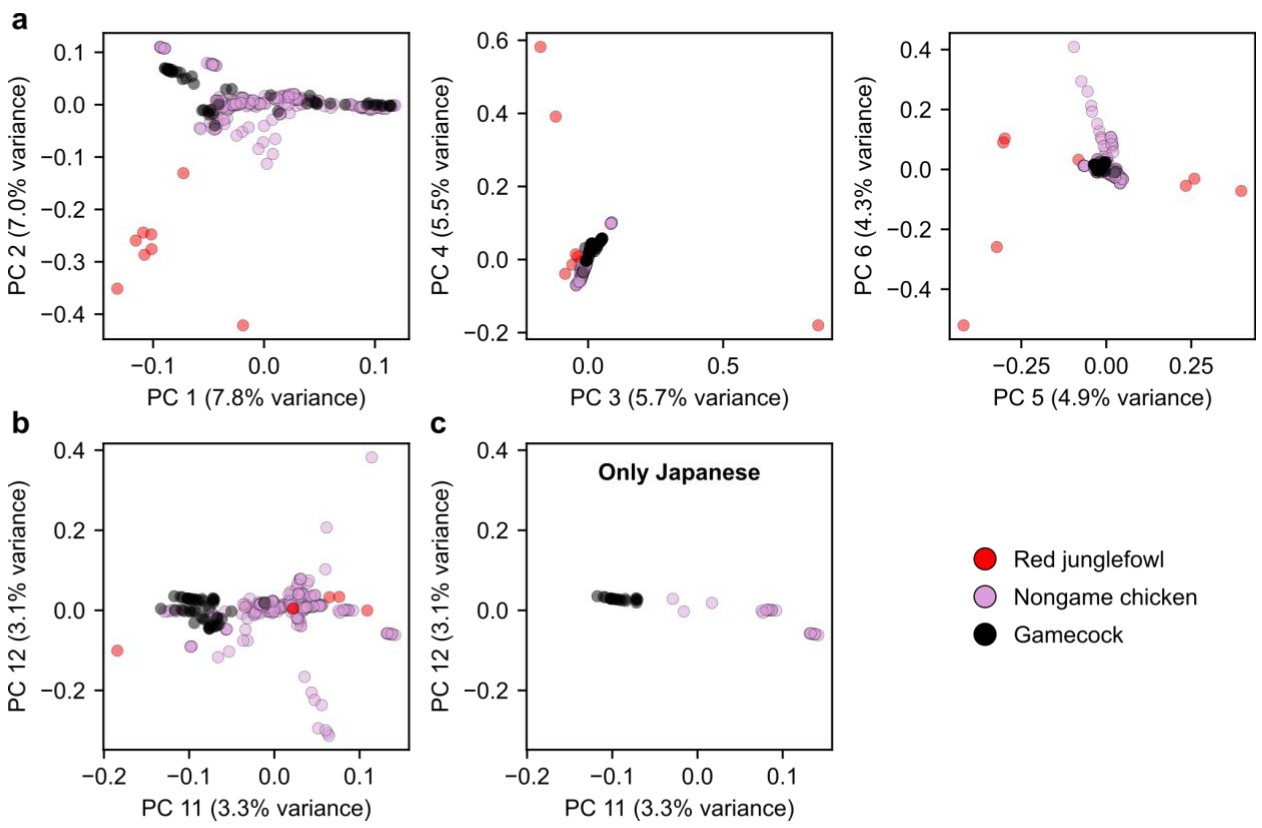

Figure S2. PCA of genetic variation in *Gallus gallus* samples.

a, PC 1 and 2 separate wild Red junglefowl from domesticated chickens (nongame chickens and gamecocks). **b,** PC 11 mostly separates chickens into nongame chickens and gamecocks. **c,** Same as **b** but including Japanese samples exclusively.

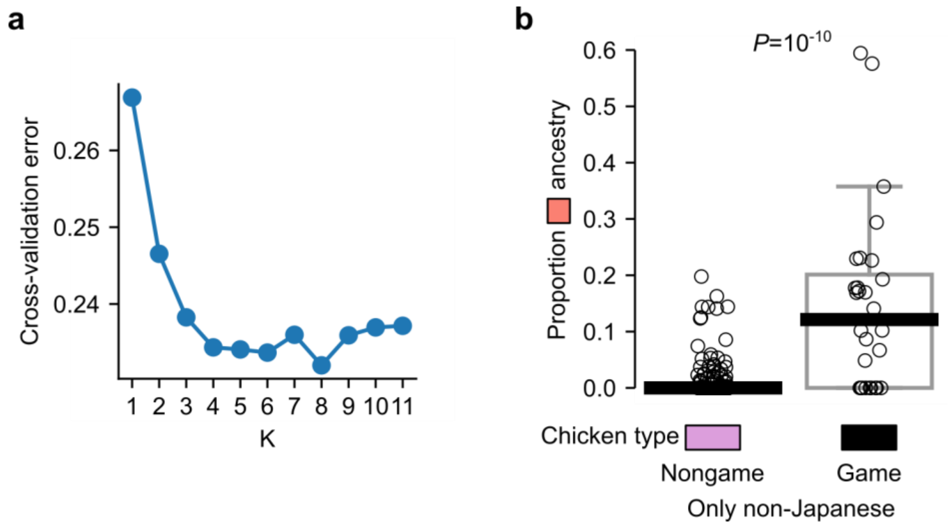

Figure S3. ANCESTRY cross-validation error and analysis without Japanese samples

a, Cross-validation error at different values of K. **b,** Proportion "salmon-colored" ancestry in samples that are not from Japan.

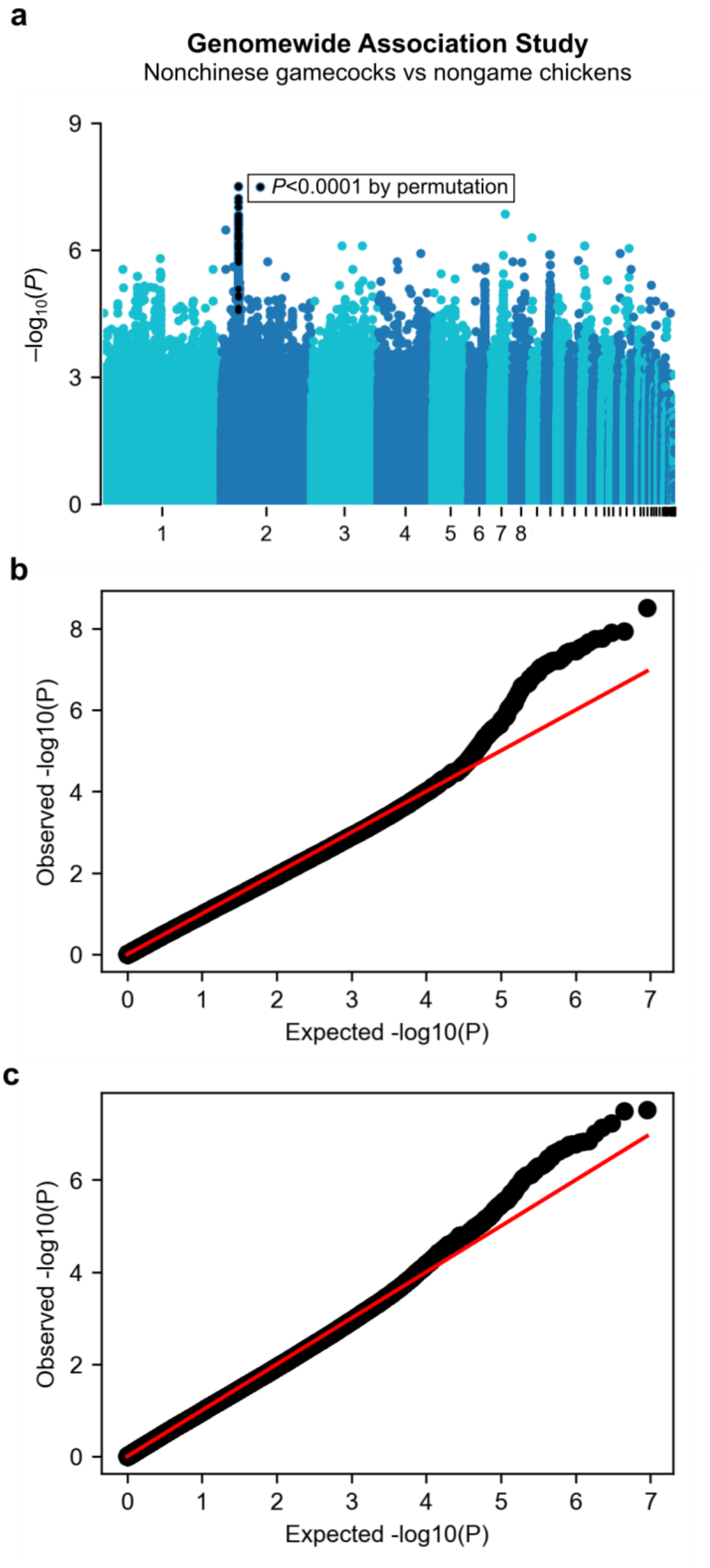

Figure S4. Genome-wide association study excluding Chinese chickens and Q-Q plots

a, GWAS of gamecocks that are not from China (n=44) vs nongame chickens (n=62). *P*-values on the y axis using genomic control. Black denotes variants with $P<10^{-4}$ by permutation; no variants outside chromosome 2 surpassed that permutation threshold. **b,** Q-Q plot of GWAS in Figure 2a. **c,** Q-Q plot of GWAS in panel **a** of this figure.

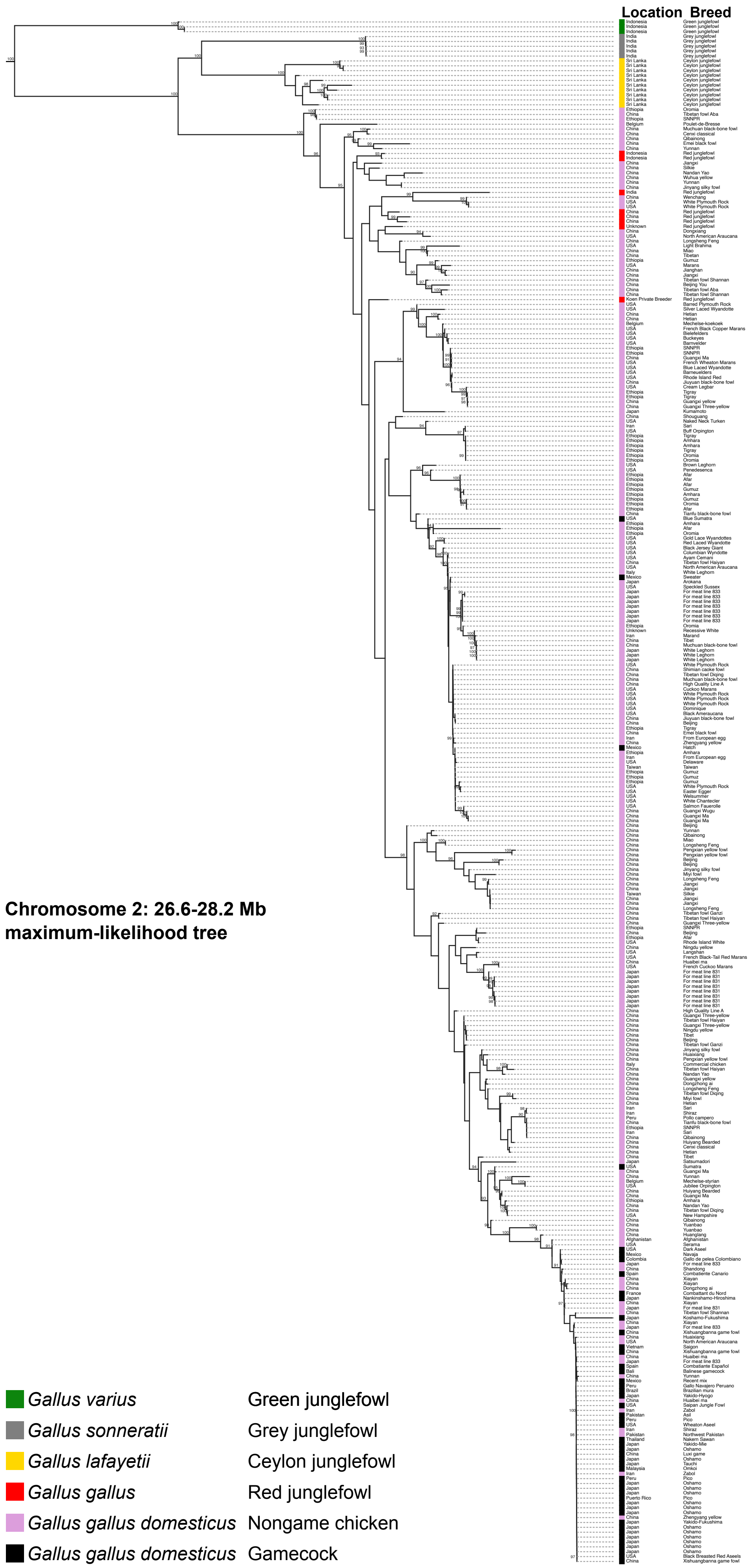
Location Breed
Chromosome 2: 26.6-28.2 Mb
maximum-likelihood tree
Gallus varius Green junglefowl
Gallus sonneratii Grey junglefowl
Gallus lafayetii Ceylon junglefowl
Gallus gallus Red junglefowl
Gallus gallus domesticus Nongame chicken
Gallus gallus domesticus Gamecock

Figure S5. Phylogenetic tree of Chromosome 2 PBS locus that distinguishes gamecocks from nongame chickens

Maximum-likelihood phylogenetic tree of Chromosome 2 locus (26.6–28.2 Mb), including all species in the junglefowl (*Gallus*) genus, as well as gamecocks and nongame chickens from around the world. The name and geographic origin of the samples is presented on the right. Bootstrap support values ≥90 are highlighted on the branches.

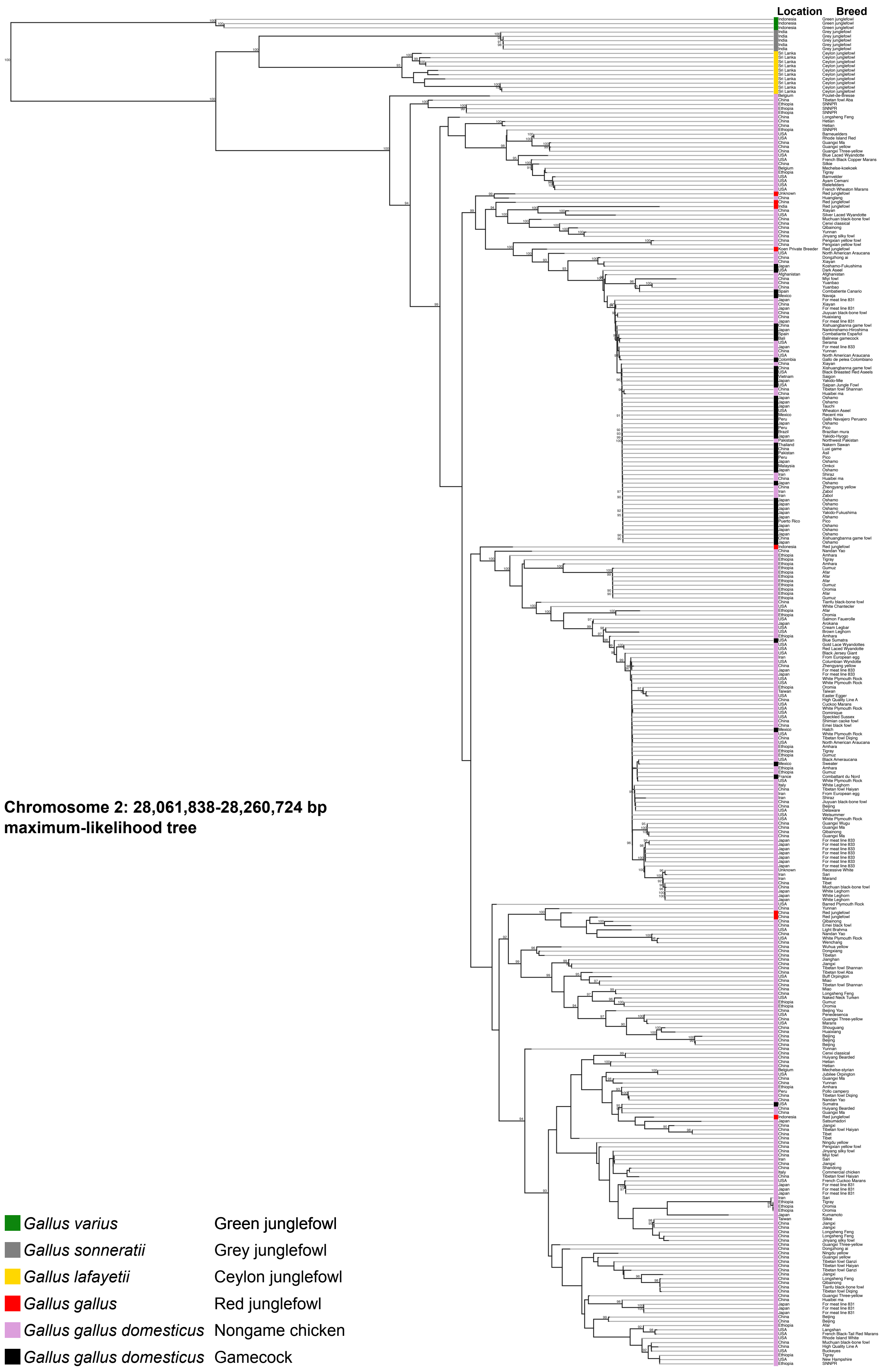
Location
Breed
Chromosome 2: 28,061,838-28,260,724 bp
maximum-likelihood tree
Gallus varius
Green junglefowl
Gallus sonneratii
Grey junglefowl
Gallus lafayetii
Ceylon junglefowl
Gallus gallus
Red junglefowl
Gallus gallus domesticus
Nongame chicken
Gallus gallus domesticus
Gamecock

Figure S6. Phylogenetic tree of Chromosome 2 GWAS locus that distinguishes gamecocks from nongame chickens

Maximum-likelihood phylogenetic tree of Chromosome 2 locus (28,061,838–28,260,724 bp), including all species in the junglefowl (*Gallus*) genus, as well as gamecocks and nongame chickens from around the world. The name and geographic origin of the samples is presented on the right. Bootstrap support values ≥90 are highlighted on the branches.

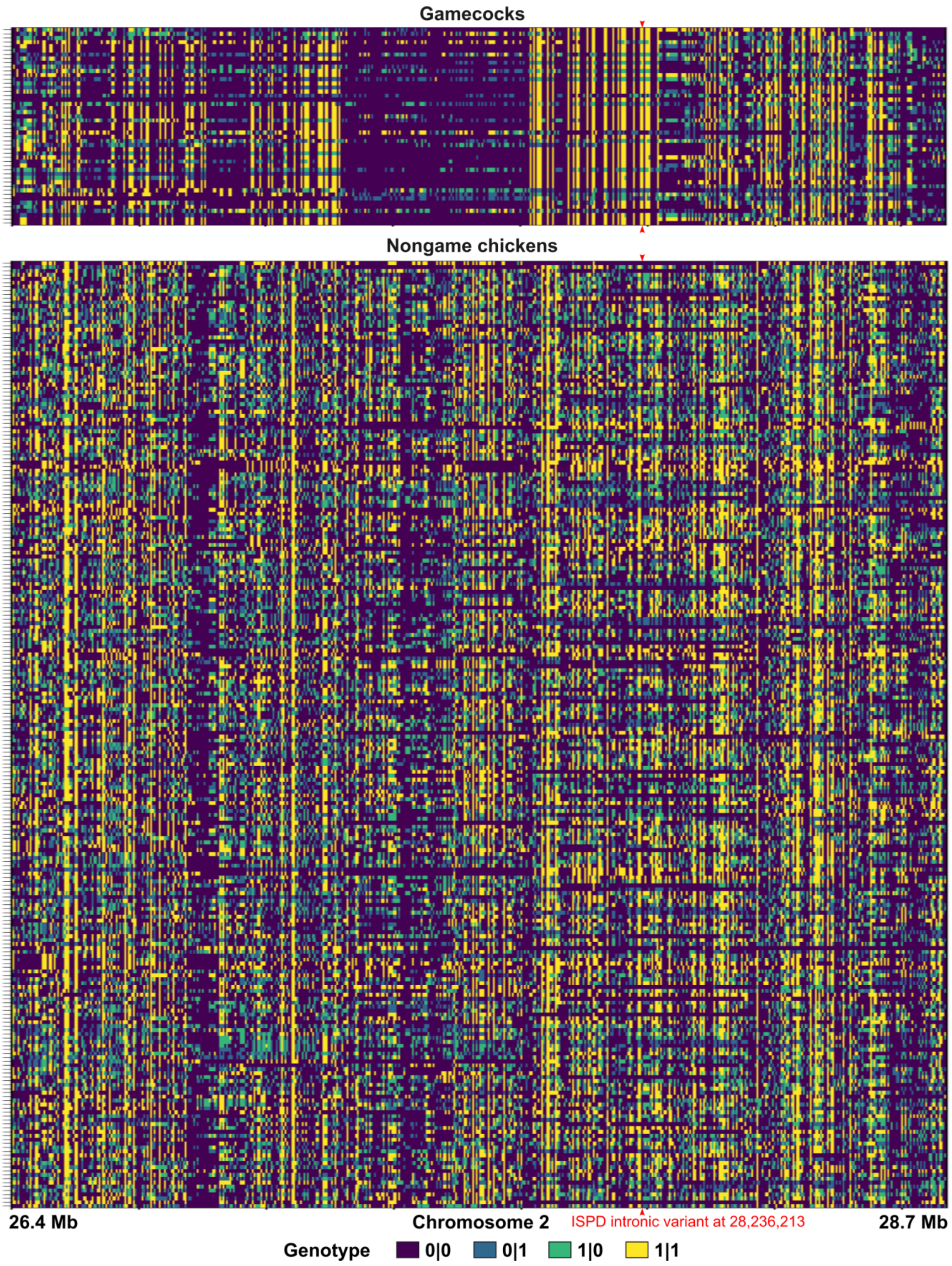

Figure S7. Haplotypes of Chromosome 2: 26.4–28.7 Mb in gamecocks and nongame chickens
